# Socio-family Factors Predictive of Adaptative Coping Post COVID-19 Pandemic in Nursing Students from a Private University* **

**DOI:** 10.17533/udea.iee.v41n2e05

**Published:** 2023-08-18

**Authors:** Luz Enith Velásquez Restrepo, Gladys Judith Basto Hernández, María Nelcy Muñoz Astudillo

**Affiliations:** 1 Master’s Nurse. Professor. E-mail: lvelasquez@areandina.edu.co. Fundación Universitaria del Área Andina, Pereira (Colombia). https://orcid.org/0000-0002-0063-7066 Fundación Universitaria del Área Andina Fundación Universitaria del Área Andina Pereira Colombia lvelasquez@areandina.edu.co; 2 Master’s Nurse. Professor. E-mail: gbasto@areandina.edu.co Fundación Universitaria del Área Andina, Pereira (Colombia). https://orcid.org/0000-0001-5732-4645 Fundación Universitaria del Área Andina Fundación Universitaria del Área Andina Pereira Colombia gbasto@areandina.edu.co; 3 Master’s Nurse. PhD candidate. Fundación CINDE/ Universidad de Manizales (Colombia). E-mail: marianelcy@gmail.com https://orcid.org/0000-0002-9310-3310 Universidad de Manizales Universidad de Manizales Colombia marianelcy@gmail.com

**Keywords:** cross-sectional studies, COVID-19, family relations, adaptation, psychological, students, nursing, socioeconomic factors, estudios transversales, COVID 19, relaciones familiares, adaptación, estudiantes de enfermería, factores socioeconómicos, estudos transversais, COVID-19, Relações familiares, adaptação psicológica, estudantes de enfermagem, fatores socioeconômicos

## Abstract

**Objective:**

To identify socio-academic and family functionality factors - communication, cohesion, and flexibility - as predictive stimuli of adaptive coping of nursing university students in the post-COVID-19 pandemic.

**Methods:**

A cross-sectional descriptive study with stratified random sampling, with participation by 416 Nursing students from a private university in Pereira (Colombia), who answered a self-completed sociodemographic characterization survey, the Olson *et al*., communication scale, FACES III scale to assess family cohesion and flexibility, and the Calixta Roy CAPS scale to assess coping and adaptation capacity. Binary logistic regression and Hosmer-Lemeshow goodness-of-fit were performed to determine predictors of success, using SPSS v.26.

**Results:**

The profiles of the participants showed a higher proportion of women (78.4%), ages between 21 and 30 years (57.5%), young people who study and work (60.1%), and those who have an academic session on Friday and Saturday (67.5%). Nursing students perceive that their families communicate efficiently and satisfactorily (85.8%), have strong cohesion with a tendency towards attachment (73.6%) and flexibility, show a tendency towards chaos (70.7%) and have adaptive coping (48.5%). The success predictors for adaptive coping were female sex (*p*=0.007), academic session Friday and Saturday (*p*=0.042), occupation, study, and work (*p*=0.026), socioeconomic strata 4.5 and 6 (*p*=0.041), good or very good communication (*p*=0.001), balanced family cohesion (*p* = 0.048), and balanced family flexibility (*p*=0.039).

**Conclusion:**

This study found that good family functionality and having adequate socioeconomic conditions were predictors of higher coping and adaptation capacity during the COVID-19 pandemic in the nursing students who participated in the study.

## Resumen

**Objetivo:**

Identificar factores socio-académicos y de funcionalidad familiar -comunicación, cohesión y flexibilidad-, como estímulos predictores de afrontamiento adaptativo de estudiantes de Enfermería en postpandemia COVID-19.

**Métodos:**

Estudio descriptivo transversal con muestreo aleatorio estratificado. Participaron 416 estudiantes de Enfermería de una universidad privada de Pereira (Colombia) que respondieron una encuesta auto diligenciada de caracterización sociodemográfica y tres escalas validadas: de Comunicación de Olson *et al.,* FACES III para valorar cohesión y flexibilidad familiar y escala CAPS de Calixta Roy para evaluar capacidad de afrontamiento y adaptación. Los factores predictores de éxito se analizaron con regresión logística binaria y bondad de ajuste de Hosmer-Lemeshow, utilizando SPSS v.26.

**Resultados:**

El perfil de los participantes mostró mayor proporción de mujeres (78.4%), edades entre 21 y 30 años (57.5%), jóvenes que estudian y trabajan (60.1%), y quienes cumplen jornada académica viernes y sábado (67.5%). Los estudiantes de enfermería perciben que sus familias se comunican en forma eficiente y satisfactoria (85.8%), tienen una fuerte cohesión con tendencia al apego (73.6%) y a la flexibilidad, muestran tendencia al caos (70.7%) y tienen afrontamiento adaptativo (48.5%). Los predictores de éxito para afrontamiento adaptativo fueron: sexo femenino (*p*=0.007), jornada académica viernes y sábado (*p*=0.042), ocupación estudia y trabajo (*p*=0.026), estratos socioeconómicos 4,5 y 6 (*p*=0.041), buena o muy buena comunicación (*p*=0.001), cohesión familiar equilibrada (*p*=0.048) y flexibilidad familiar equilibrada (*p*=0.039).

**Conclusión:**

En este estudio se encontró que la buena funcionalidad familiar y tener adecuadas condiciones socioeconómicas fueron predictores de mayor capacidad de afrontamiento y adaptación durante la pandemia COVID-19 en los estudiantes de enfermería que participaron en el estudio.

## Resumo

**Objetivo:**

Identifico os fatores socioacadêmicos e a funcionalidade familiar -comunicação, coesão e flexibilidade-, como estímulos preditivos do enfrentamento adaptativo de estudantes de Enfermagem no pós-pandemia de COVID-19.

**Métodos:**

Estudo descritivo transversal com amostragem aleatória estratificada. Participaram 416 estudantes de enfermagem de uma universidade privada da cidade de Pereira (Colômbia), respondendo a uma pesquisa autopreenchida de caracterização sociodemográfica e três escalas validadas: Comunicação de Olson *et al.,* FACES III para avaliar a coesão e flexibilidade familiar e a escala CAPS de Calixta Roy, para avaliar a capacidade de enfrentamento e adaptação. Os preditores de sucesso foram analisados com regressão logística binária e Hosmer-Lemeshow, usando SPSS v.26.

**Resultados:**

Os perfis dos participantes mostraram maior proporção de mulheres (78.4%), com idades compreendidas entre os 21 e os 30 anos (57.5%), jovens que estudam e trabalham (60.1%) e que cumprem o horário letivo de sexta-feira a sábado (67.5%). Os estudantes de enfermagem percebem que suas famílias se comunicam de forma eficiente e satisfatória (85.8%), têm forte coesão com tendência ao apego (73.6%) e flexibilidade, apresentam tendência ao caos (70.7%) e têm enfrentamento adaptativo (48.5%). Os preditores de sucesso para enfrentamento adaptativo foram: sexo feminino (*p*=0.007), jornada acadêmica sexta e sábado (*p*=0.042), ocupação, estudo e trabalho (*p*=0.026), estrato socioeconômico 4.5 e 6 (*p*=0.041), boa ou comunicação muito boa (*p*=0.001), coesão familiar equilibrada (*p*=0.048) e flexibilidade familiar equilibrada (*p*=0.039).

**Conclusão:**

Neste estudo, verificou-se que a boa funcionalidade familiar e ter condições socioeconômicas adequadas foram preditores de maior capacidade de enfrentamento e adaptação durante a pandemia de COVID-19 nos estudantes de enfermagem que participaram do estudo.

## Introduction

As a result of constant social transference derived from the globalization of the economy, most social formations have incorporated customs, forms, and organizational models that have transformed the traditional notion of family. Since its origins, the family has been conceived as a social cell comprised by individuals with some degree of kinship, as the primary place where the social risks of its members are shared and managed;^1^ the idea of family has gone through different moments, consistent with the historical-social development of the peoples, and in each one, the concept has been configured from the different disciplines, from the established hegemonic guidelines,^2^ until it is currently read as a diverse, complex, dynamic reality that moves to the rhythm of the social order, with the multiplicity of conflicts and tensions that constitute it..^3^ In this sense, it must be considered that each family has its own dynamics and functionality, being that, as proposed by Palacio,^3^ there are five issues that cross it, that are tied in the intimate space of the family and make its identification complex: sexuality, procreation, cohabitation, survival and coexistence; these issues, analyzed in numerous manuscripts within the context of the COVID-19 pandemic^4^^-^^6^ force a concept of family as flexible as the institution itself. Seeking to respond to the complexity conditions described, for the purpose of this study, the interdisciplinary concept of family proposed by Oliva and Villa will be used: *“*The family is a group of two or more people who live as a spiritual, cultural, and socioeconomic unit, which, even without physically living together, share psycho-emotional and material needs, objectives and common development interests, from different aspects whose priority and dynamics belong to their free will: psychological, social, cultural, biological, economic, and legal.” ^2^:^17^

The cohabitation and subsistence capacities of families in all of humanity were widely exposed within the framework of the COVID-19 Pandemic; anxiety, fear, uncertainty, pain, sensations, emotions and feelings found in the reduced spaces of physical confinement, shook, stripped, and made visible the intimacy of their coexistence in the social space; their private world became a public arena for agreements and disputes; family strengths and weaknesses surfaced.^7^^-^^9^ Numerous authors have characterized the families during times of pandemic. Their writings highlight six analytical categories: (i) Coexistence: modification in family interactions, social interdependence;^7^^,^^8^ (ii) Reinvention of lifestyles;^9^ (iii) Resignification of values;^9^^,^^10^ (iv) Affective communication: emotion management, expression of affection;^7^^-^^10^ (v) Support networks: spirituality enhancement, social and family support,^7^^,^^10^ and (vi) Adaptation to the situation with change of limits, roles, and routines. ^7^^,^^10^ All these categories guide toward the importance of affective communication, family cohesion, flexibility in roles and responsibilities, as well as deployment of different coping mechanisms in subfamily systems, to survive and stay healthy. ^8^

Within this context, family health gains strength as one of the performance pillars of the Nursing profession; ethical, legal, and social commitment exists with the comprehensive care of the family. Several nursing theorists have approached the family as a subject of care.^11^ Considering the impact of the COVID-19 Pandemic on higher education in health,^12^ and specifically in Nursing,^13^ this study explored *a-posteriori* the coping and family adaptation mechanisms used by nursing students during the crisis period, for which elements were taken from Calixta Roy’s Adaptation Model.^14^ According to this Nursing theorist, the human system is a set of interdependent and connected parts in function of a whole and adaptation is a process and result through which these human systems - capable of thinking, feeling, and interacting - choose to integrate with their environment. Roy considers that people are in permanent interaction with their environment; all kinds of *focal*, *residual*, and *contextual* stimuli enter dynamically into the human adaptive system that trigger two central coping processes: the *regulator* (controls internal processes related with physical-physiological needs) and the *cognator* (regulates self-concept, role function, and interdependence). Depending on the responses triggered, the level of adaptation is assessed; to specifically identify individual nursing intervention needs, Roy differentiates five coping factors: F1: Recursive-centered; F2: Focused physical reactions; F3: Alert process; F4: Systematic processing, and F5: Know and relate. ^14^

Taking Calixta Roy's thought as a reference for understanding, the aim is to explore the strength of the relationship between coping and adaptation capacity by nursing students amid the crisis derived from the COVID-19 Pandemic, and the residual and contextual stimuli from their environment: from residual type: some socio-academic characteristics and, contextually, the family functionality. According to Callupe *et al.*,^15^ the level of family functioning predicts the degree of resilient coping. Said family functionality is studied from the perspective by David Olson *et al.*, embodied in the Circumplex Model of Marital and Family Systems. To implement this Model, the authors developed in 1980 the Family Adaptability and Cohesion Evaluation Scales (FACES); it has been modified to improve its psychometric properties, thus FACES (1980), FACES II (1982), FACES III (1985), and FACES IV (1990).^16^^-^^19^ The model is centered on the three principal dimensions of the family system: cohesion, flexibility, and communication. Cohesion refers to the support that family members give each other, the mutual commitment to the well-being of another, that is, how united or separated they are from the rest of the family.^16^^,^^17^ Flexibility refers to the amount of change the family experiences regarding leadership, control, discipline, negotiation styles; that is how stability and change are balanced.^16^^,^^17^ Communication is understood as the capacity to transfer information about feelings, needs, and emotions among the family members and prioritizes active listening and attachment, with balanced family systems being those that tend to be more functional compared to unbalanced systems.^18^^-^^20^

This initial evaluation is constituted as a starting point to facilitate greater levels of adaptation by strengthening family coping mechanisms. The aim of this study was to identify socio-academic and family functionality factors, like communication, cohesion, and flexibility, as predictive stimuli of adaptative coping by university nursing students, during post COVID-19 Pandemic.

## Methods

Cross-sectional descriptive study conducted with a population of 1,360 nursing students from a private higher education institution in the city of Pereira in Risaralda, Colombia, in 2021. The sample was random stratified by academic semester, with 95% reliability and 4% precision error; under these conditions, the minimum sample size was 416 students. 

Four instruments were used to collect the information: 

Sociodemographic characterization survey that included 10 variables: age, sex, marital status, number of children, living with whom, household strata, occupation, semester in course, scheduled academic session (A: Monday to Thursday and B: Friday and Saturday), and department of residence. All the variables were categorical and were dichotomized for the multivariate analysis.

Communication scale by Olson *et al*., created in 1982 by Barnes and Olson^17^ and has been modified up to the 10-item version in a 1 to 5 Likert scale, formulated in positive, so that, the higher the score obtained, the higher the level of communication. The psychometric properties for its application were analyzed in the Peruvian Institute of Psychological Guidance in 2016, where an internal consistency index of Cronbach's alpha α=0.887 was found.^19^ The version used in this study was adapted for Latin America and validated in 2017 with Chilean adult population; a factorial solution of two constructs was found with internal consistencies of 0.895 and 0.854 for each construct.^18^ This study reiterated the high reliability of this scale with a Cronbach's alpha of 0.90.

FACES III scale. This version evaluates cohesion and family flexibility; it has 20 items, in a 1 - 5 Likert scale. Odd items value cohesion and even items value flexibility. There are four cohesion levels: detached (disconnected), separate, connected (united) and entangled (amalgamated); the balanced levels are separate and connected. There are four flexibility levels: chaotic, flexible, structured, and rigid; the balanced levels are flexible and structured. The higher the score of the scales, the greater cohesion or flexibility. Proportions permit identifying needs for intervention.^17^ Crossing cohesion and flexibility levels in a 4x4 table makes possible 16 family typologies according to functionality, of which four are extremely dysfunctional, eight are moderately functional [medium range] and four are balanced functional. FACES III has been validated in several Latin American countries; the cohesion scale showed high reliability with Cronbach’s alpha between 0.70 and 0.90; the flexibility scale showed moderate reliability with Cronbach’s alpha between 0.65 and 0.70.^16^^,^^7^^,^^21^^-^^23^ For this study, application of FACESIII in nursing students showed high reliability, with Cronbach’s alpha of 0.92.

The CAPs scale by Calixta Roy, validated for Colombia by Sarmiento *et al.* ^24^ permits evaluating coping and adaptation capacity. It comprises 33 items, of which items 4, 8, 9, 14, 15, 16, 22, 23, 27, and 31 are inverted. According to Roy, coping can be effective or ineffective; the largest proportions by coping factor in its high, mean or low measurement permit identifying intervention needs. To interpret the scores, it is considered that a higher score in the scale means a higher adaptation level; it can be integrated, compensatory, or committed. Univariate and bivariate statistical analyses were performed, considering coping and adaptation capacity as dependent variable. A value of *p* < 0.05 was considered significant. To know the success prediction factors, binary logistic regression and Hosmer-Lemeshow goodness-of-fit were performed, using SPSS v.26. 

Ethical considerations. For data collection, sensitization was conducted with the selected students, after signing the informed consent, each scale was explained and instruments for self-completion were distributed. Participant anonymity and the right to withdraw from the process were preserved. Approval by the institutional ethics committee was obtained according to the Minutes of May 08, 2022.

## Results

The higher education institution selected - and specifically - the Nursing Program gathers students from throughout the Colombian southwest. This research had the participation of 416 students from the Nursing Program, ranging in age between 17 and 46 years; the ages were categorized from the survey. As seen in Table 1, the sociodemographic profile showed predominance of students under 21 to 30 years of age, of female sex, single marital status, and without children. The majority of those surveyed lived with their family nucleus. Their households are in socioeconomic strata I-II and III. It was noted that a high proportion of students worked and studied, were matriculated in academic session schedule B for Friday and Saturday and came to study from Valle del Cauca and Risaralda.

**Table 1:** Sociodemographic characterization of the 416 nursing students

Variable	Values	Frequency	Percentage
Age group	Up to 20 years	74	17.8
	From 21-30 years	239	57.5
	From 31-40 years	90	21.6
	Over 40 years	13	3.1
Sex	Female	326	78.4
	Male	90	21.6
Occupation	Study	166	39.9
	Study/ work	250	60.1
Socioeconomic level	Strata I-II	247	59.4
	Strata III-IV	161	38.7
	Strata V-VI	8	1.9
Number of children	0	280	67.3
	1	84	20.2
	2	46	11.1
	3 or more	6	1.4
Type of cohabitation	Family nucleus	327	78.6
	Couple	56	13.5
	Alone	13	3.1
	Friend	11	2.6
	Other	9	2.1
Marital status	Married	37	8.9
	Divorced	3	0.7
	Single	276	66.3
	Common law	100	24.0
Semester in course	From 1 to 4	178	42.8
	From 5 to 8	238	57.2
Academic session	Monday to Thursday	135	32.5
	Friday and Saturday	281	67.5
Department of origin	Valle del Cauca	165	39.7
	Risaralda	115	27.6
	Other	136	32.7

### Communication

It is taken as an axiomatic assumption that all social relationships are built, acquire meaning and trajectory from the forms of interaction. Communication permits families to construct their social world from the interactions and shared meanings among their members, during the time that they coexist and are maintained as a whole. According to the findings of this study, students perceive that communication between good and very good predominates in 85.8% of their families, which, in addition, is effective and satisfactory. Table 2 shows the proportional distribution of the scores obtained in the scale, the means, and the standard deviation (SD) per item. There is greater dispersion in items 9 and 5 that focus on everyday difficulties to achieve excellent communication when moods are altered.

**Table 2:** Family communication in 416 nursing students

Items	Communication scale. Proportions					Mean*	SD
	Very deficient	Deficient	Regular	Good	Very good		
1. My family members are satisfied with the way we communicate	3.1	5.5	9.9	45.2	36.3	4.06	0.98
2. My family members are very good at listening	4.1	6.7	15.1	40.6	33.5	3.93	1.05
3. My family members express affection to each other	3.6	5.5	8.7	31.7	50.5	4.20	1.04
4. My family members are capable of asking each other what we want	3.1	5.8	9.9	38.9	42.3	4.12	1.01
5. My family members can calmly discuss our problems	7.2	11.8	14.4	38.9	27.7	3.68	1.20
6. My family members discuss our ideas and beliefs	7.5	6.5	10.6	41.6	33.8	3.88	1.16
7. When family members inquire about something, they receive honest answers	3.4	4.6	7.0	39.9	45.1	4.19	0.98
8. Family members try to understand the feelings of other members	4.8	6.7	10.1	37.5	40.9	4.03	1.10
9. When family members are angry, they rarely say negative things to each other	9.9	13.0	20.2	35.3	21.6	3.46	1.23
10. Family members express their true feelings	4.8	5.0	10.8	30.3	49.1	4.14	1.10
Total	0	3.4	10.8	33.2	52.6	39.60	8.30

* 1: minimum, 5 maximum

### Family functionality

Family cohesion. A functional, healthy family is characterized by the effective equidistance between attachment and detachment. Extreme positions can perjudicar the family wellbeing. Excessive attachment restricts freedom and promotes mutual dependence, while extreme freedom leads to loneliness and lack of belonging. It is necessary to have affection, union, trust, support, and for the independent development of each of its members to be encouraged. According to the findings, among the families of the students, the united and the separated ones predominate (57.3%); in the former there is a high level of emotional closeness and the latter are characterized by moderate cohesion, it is not as extreme as divided families; in synthesis, they have a strong bond, with a tendency to attachment. If the family members request it, support is offered, but not all participate in the decision making. Although there is an opening to share time with friends of family members, people prefer to interact with the closest relatives and enjoy family gatherings. Table 3 shows the frequencies of the scores obtained in the scale, the means, and standard deviation (SD) per perceived behavior.

**Table 3:** Family cohesion in 416 nursing students

Dimensions	Perceived behaviors	Cohesion scale. Percentages					Mean *	SD
		1. Lower cohesion	2	3	4	5. Higher cohesion		
Emotional bonding	11. My family members feel very united	3.2	4.2	12.7	21.7	58.2	4.28	1.04
	19. In our family, the sense of family union is very important	1.9	2.6	6.7	17.1	71.7	4.54	0.88
Support	1. My family members ask for help when they need it	0	1.9	10.6	22.6	64.9	4.50	0.76
	17. In my family we consult each other when we are going to make a decision	6.3	6.3	20.4	30.8	36.2	3.85	1.16
Family limits	5. We prefer to associate with the closest relatives	5.5	8.2	20.7	37.0	28.6	3.75	1.12
	7. The members of our family feel closer to each other than to other people who are not part of our family	8.9	10.1	17.8	28.8	34.4	3.70	1.28
Time and friends	3. Friends of other family members are accepted	0.7	3.4	16.1	38.2	41.6	4.20	0.86
	9. My family members like to spend our free time together	2.4	5.0	14.7	26.7	51.2	4.19	1.02
Interests and Recreation	13. When our family carries out an activity, we all participate	4.8	6.5	16.6	31.3	40.8	3.97	1.12
	15. It is easy to think of activities we can carry out as a family	2.6	6.5	18.8	30.3	41.8	4.02	1.05
Family cohesion scale							40.93	6.6

* 1: minimum, 5 maximum

Family flexibility. A functional family keeps healthy relations in negotiation processes among its members, with respect to discipline, roles, and leadership styles; their flexibility accounts for their capacity to change, yield or impose points of view when faced with solving everyday problems due to regulatory crises. The continuum transits between chaos and rigidity. Extremes are not healthy; chaos is derived from the lack of foresight and lead to non-regulatory crises; on the other hand, rigidity stems from excesses in hierarchy and control. Most of the families of the students were classified in the scale’s intermediate levels: flexible and structured (63%); however, a tendency towards chaos is noted, this observation is based on the lack of leadership, no visible head exists to summon the attention and respect of all the members; there is difficulty in assigning responsibilities and excessive patternal control in decision making (Table 4).

**Table 4:** Family flexibility in 416 nursing students

Indicators	Perceived behaviors	Flexibility scale. Percentages					Mean	SD
		1. Lower flexibility	2	3	4	5. Higher flexibility		
Leadership	6. There are several people who rule in our family	17.5	19.0	29.3	20.3	13.9	2.94	1.28
	18. It is difficult to know who rules in our family	47.8	16.8	12.0	14.5	8.9	2.20	1.39
Discipline	4. When establishing rules of discipline, the opinion of the children is taken into account	2.6	6.5	22.9	32.7	35.3	3.92	1.03
	10. Parents and children talk about punishment	13.2	11.9	21.9	27.9	25.1	3.40	1.32
Control	2. When a problem arises, the opinions of the children are taken into account	0.4	4.8	16.4	30.1	48.3	4.20	0.90
	12. Children make decisions in our family	28,8	19,7	31,2	10.8	9.5	2.53	1.27
	8. Faced with different situations, our family changes the way it handles them	6.7	7.6	30.1	31.3	24.3	3.60	1.12
Roles and rules	14. In our family, norms or rules can be changed	11.2	16.8	40.9	19.5	11.6	3.04	1.12
	16. Among the family members, we take turns on the responsibilities of the house	3.4	6.7	19.5	29.8	40.6	3.98	1.08
	20. It is easy to say what task each family member has	30.0	21.9	22.8	13.5	11.8	2.55	1.35
Flexibility scale							32.24	6.42

* 1: minimum, 5 maximum

**Figure 1:**
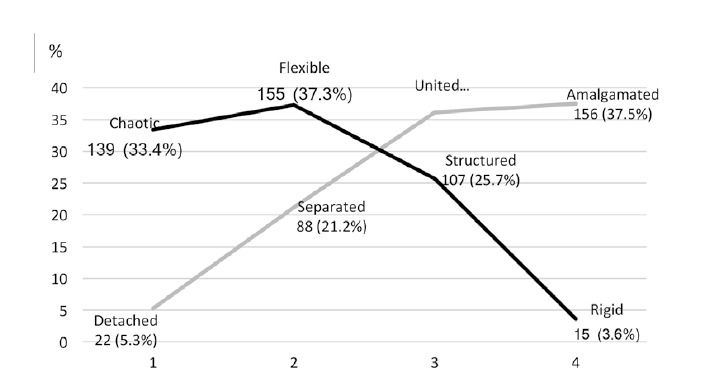
Cohesion and flexibility in families of 416 nursing students

The cohesion and family flexibility scales, as established by the model proposed by Olson *et al*., permit classifying family typologies according to functionality; the continuum of each scale transits between two poles where the families with extreme dysfunctionality are located, with functional families remaining in the middle. The findings from this study are recorded in Figure 1. The cohesion scale shows tendency to attachment, when adding the united and amalgamated typologies (73.6%), while that of flexibility is inclined toward chaos, when joining the flexible and chaotic typologies (70.7%). The similarity in the proportions for each value of the scales permits interpreting the families of the nursing students as family systems with balance in tension, a fact that, together with the perception of very good and good communication among its members, allows explaining - in part - the findings on family functionality, which are described ahead.

Family functionality. From the combination of the categories of cohesion and flexibility, as proposed by the Circumplex Model for the Interpretation of FACES III in the standard updated for 2017 by Bazo-Alvarez *et al.*,^24^ 16 family typologies emerge. The proportions found for each typology are registered in Table 5. The highest proportion corresponded to functional families (41.3%), followed by the middle range (37.5%) and, lastly, extreme dysfunctionality (21.2%).

**Table 5:** Functionality levels and Family typologies in students

Functionality levels	Family typologies	Frequency	Percentage
Balanced Functional	Separate / Flexible	35	8.4
	United / Flexible	61	14.7
	Separate / Structured	38	9.1
	United / Structured	38	9.1
	Total	172	41.3
Middle Range	Detached / Structured	12	2.9
	Detached / Flexible	2	0.5
	Separate / Rigid	6	1.4
	United / Rigid	4	1.0
	Entangled/ Flexible	57	13.7
	Entangled / Structured	19	4.6
	United / Chaotic	47	11.3
	Separate / chaotic	9	2.2
	Total	156	37.5
Extreme dysfunctionality	Entangled / chaotic	80	19.2
	Detached / chaotic	3	0.7
	Detached / Rigid	5	1.2
	Entangled / Rigid	0	0
	Total	88	21.2

Coping and adaptation. As seen in Table 6, the factors with the highest percentage in high coping capacity were F1 Centered recursive, F4 Systematic processing, and F5 Know and relate. These findings permit characterizing creative families, who seek results based on their knowledge, have a methodical capacity to solve problems with concrete actions, and use effective social interaction strategies. In over half the students, adaptative coping was found at compensatory level.

**Table 6:** Coping factors and levels of family adaptation (CAPs) in 416 university nursing students

Coping factors	Coping capacity						Mean per factor	Items per Factor	Mean*	**SD**
	High		Medium		Low					
	Frequency	Percentage	Frequency	Percentage	Frequency	Percentage				
Factor 1. Centered recursive	165	39.7	245	58.9	6	1.4	14.02	7	2.0	(0.00)
Factor 2. Physical and focused reactions	93	22.2	301	72.5	22	5.3	20.83	12	1.7	(4.24)
Factor 3. Alert process	78	18.8	274	65.9	64	15.4	11.62	6	1.9	(0.70)
Factor 4. Systematic processing	160	38.5	243	58.4	13	3.1	6.32	3	2.1	(0.70)
Factor 5. Know and relate	150	36.1	257	61.7	9	2.2	9.93	5	2.0	(2.12)
Adaptative Coping - Level										
Integrated	87	20.9							76.49	(6.36)
Compensatory	224	53.8							62.92	(6.36)
Committed	105	25.3							50.9	(2.83)

* Nº items Scale 0 to 3

### Relation between sociodemographic variables and components of family dynamics

Significant relations were sought between the dichotomized sociodemographic variables (SD) and the family dynamics variables of communication, cohesion, flexibility, and adaptative coping. To dichotomize the variable of adaptative coping, the total score of the CAPs scale was used. The scale range used is from 0 to 99; in medium coping, scores are between 57 and 70. The midpoint corresponds to 63. A score above 63 was considered adaptive coping. 

No significant relations were found with the variables of marital status, family coexistence, and socioeconomic level. Family cohesion was not associated significantly with the variables analyzed in dichotomous manner. Table 7 registers the results of the chi squared test with *p* < 0.05 for each of the significant relations.

- Communication was associated significantly with variables of age, having children, occupation, academic session and semestre: of all the students with good or very good communication, the highest proportions were found in: those under 30 years of age ( 72.5%); in those without children (64.7%), in those who study and work (64.1%), those attending academic sessions on Friday and Saturday (71.7%) and those who are in the 5^th^ to 8^th^ semesters (59.7%).
- Family flexibility was associated significantly with variables of sex, academic session, and department of residence: of all the students with functional families (flexible or structured), the highest proportion corresponded to females (82.8%), to those studying in the academic sessions on Friday and Saturday (64.1%).
- Adaptative coping was associated significantly with age group, sex, occupation, scheduled academic session, and semester: of all the students with adaptative coping, the highest proportions were found in: those under 30 years of age (70.1%), females (75.4%), who study and work (66.8%), who are registered in 5^th^ to 8^th^ semesters of the carreer (62.7%), and those matriculated in the academic sessions on Friday and Saturday (75%).

**Table 7:** Significant associations between dichotomized sociodemographic variables and communication, flexibility and adaptative coping in 416 nursing students

Variable	Values	Frequency	Percentage	Communication*	Flexibility*	Adaptative coping*
Age group	< 30 years	313	75.2	0.001		0.001
	30 years and more	103	24.8			
Sex	Female	326	78.4		0.004	0.022
	Male	90	21.6			
Has children	Yes	136	32.8	0.003		
	No	280	67.2			
Occupation	Study and work	250	60.1	< 0.001		0.001
	Study	166	39.9			
Scheduled academic session	Monday to Thursday	135	32.5	<0.001	0.050	< 0.001
	Friday and Saturday	281	67.5			
Semester in course	From 1^st^ to 4^th^ (initial)	178	42.8	0.013		0.002
	From 5^th^ to 8^th^ (final)	238	57.2			

* (^2^ test with *p* < 0.05

### Predictive factors of adaptative coping

A binary logistic regression model was applied to estimate the likelihood ratio of each variable against the dependent variable: adaptive coping. From the already-known observations, the model used revealed success of 78.5% to predict relations. A simulation of the model assigning a value of one to communication, assuming that all students had good or very good family communication, yields a probability of 0.86% that agreement is affected by this variable. Table 8 shows the variables with statistical significance in the model. The success predictive factors for adaptative coping by nursing students, during COVID-19 post-pandemic were the *residual stimuli:* female sex, scheduled academic session on Friday and Saturday; occupation study and work, belonging to socioeconomic strata 4, 5, and 6; and for the *contextual stimuli*: all the dimensions of family functionality: good or very good communication, balanced cohesion and flexibility.

**Table 8:** Binary logistic regression model for adaptative coping as dependent variable

Variables	B	Standard error	Wald	GL	*p*-value*	Exp(B)	95% CI for Exp(B)	
							Lower	Upper
Female sex	-0.794	0.293	7.342	1	0.007	0.452	0.255	0.803
Schedule B: Friday and Saturday	-0.624	0.306	4.147	1	0.042	0.536	0.294	0.977
Occupation: Study and work	0.276	0.281	0.964	1	0.026	1.318	0.759	2.287
Strata SE: IV-V-VI	1.197	0.614	3.806	1	0.041	3.310	0.994	11.019
Communication Very good or good	-1.058	0.313	11.426	1	0.001	0.347	0.188	0.641
Cohesion United or Separate	0.437	0.231	3.592	1	0.048	1.548	0.985	2.434
Flexibility Flexible or structured	-0.495	0.240	4.263	1	0.039	0.610	0.381	0.975
Constant	4.403	3.509	1.675	1	0.603	0.657		

* Hosmer-Lemeshow test: (^2^ = 7.357, GL = 8, *p* = 0.499

## Discussion

Coherent with Roy’s Adaptation model, the study participants received focal stimuli from their environment**,** mostly considered negative, like permanent reports on the spread and lethality of the virus, deaths of thousands of people - some close or known to them, permanent use of protection measures, forced confinement, forced transit towards the use of virtual learning environments and others, related in the literature. This study analyzed the contextual stimuli referring to family functionality and found that all are quite significant for coping success, but only 41.3% of the families were classified with balanced functionality. Likewise, the socio-academic residual stimuli, considered decisive factors in the adaptation process, did not predominate in the study population**.**

The post-pandemic period is a time of family adaptation crisis; although families are demonstrating great effort to maintain a balanced functionality, variables like socioeconomic situation, female sex condition, and academic-work stress expose nursing students to negative socio-familial stimuli that explain - in part -extreme dysfunctionality in 21.2% of their families, and compromised level of adaptation in 25.3% of the students. Effective communication, which shows satisfaction, listening capacity, expression of affection, interest in the other, mutual support within the family is reaffirmed as the principal component of family functionality for coping success during moments of crisis. It the study by Garcés *et al.,*^8^ family communication, in significant manner, was established as a predictor of perceived stress; offensive communication, linked to health concerns and problems in family life, increases high negative stress up to 37.9% of the total variance. In turn, Araújo *et al.,*^25^ found that a good level of communication among university adolescents and their parents predicts enjoyment and time spent on joint recreational activities. 

Cohesion, in our study, showed balanced families, favorable for family functionality; in other contexts, it revealed variations, according with the specific conditions of the dynamics analyzed. The study by Lebow ^26^ highlighted the stable coexistence of the couple as a protective factor to control negative stress. Lacomba *et al.,* ^27^ found relationships among family support, development of emotional regulation strategies, and resilience during moments of crisis. Robles *et al*.,^28^ exposed family conflicts related with mourning, violence, invasion of privacy, anxiety, stress, and economic hardship with direct impact on tuition financing and student academic performance. 

Flexibility in our study scored for family balance, however, the means obtained in the items were very low and these favored the presence of families with extreme dysfunction. Several studies showed greater capacity to balance stability and change; Aponte *et al*.,^29^ observed that family members committed to decision making and obligations of household maintenance; Callupe *et al*.,^15^ found significant correlations among flexibility, emotional bonding, family functioning, and resilient coping. 

This work concludes that balanced family functionality, good family communication, cohesion, and family equilibrium, as well as good socioeconomic conditions predict adaptative coping in the nursing students who participated in the study during times of COVID-19 post-pandemic. In this group it is necessary to strengthen family cohesion, seeking spaces for more frequent approaches when decisions are to be made involving the entire group and promoting participation by other close family members in joint activities. It is, likewise, important to strengthen family flexibility with respect to leadership, discipline, control, roles, and rules.

For professional nursing care, it is important to bear in mind that each family has a different Dynamic and specific way of coping with different stimuli; hence, family functionality should be examined in comprehensive manner, for which, Olson’s Circumplex Model is a good theoretical-methodological alternative.
